# Reproducibility of continuous glucose monitoring results under real-life conditions in an adult population: a functional data analysis

**DOI:** 10.1038/s41598-023-40949-1

**Published:** 2023-08-26

**Authors:** Marcos Matabuena, Marcos Pazos-Couselo, Manuela Alonso-Sampedro, Carmen Fernández-Merino, Arturo González-Quintela, Francisco Gude

**Affiliations:** 1 Research Methods Group (RESMET), Health Research Institute of Santiago de Compostela (IDIS), Santiago de Compostela, Spain; 2 Department of Psychiatry, Radiology, Public Health, Nursing and Medicine, University of Santiago de Compostela, Santiago de Compostela, Spain; 3 Network for Research on Chronicity, Primary Care, and Health Promotion (RICAPPS-ISCIII), Santiago de Compostela, Spain; 4 A Estrada Primary Care Center, A Estrada, Spain; 5 Internal Medicine Department, University Hospital of Santiago de Compostela, Santiago de Compostela, Spain; 6 Concepción Arenal Primary Care Center, Santiago de Compostela, Spain

**Keywords:** Endocrinology, Health care, Medical research

## Abstract

Continuous glucose monitoring systems (CGM) are a very useful tool to understand the behaviour of glucose in different situations and populations. Despite the widespread use of CGM systems in both clinical practice and research, our understanding of the reproducibility of CGM data remains limited. The present work examines the reproducibility of the results provided by a CGM system in a random sample of a free-living adult population, from a functional data analysis approach. Functional intraclass correlation coefficients (ICCs) and their 95% confidence intervals (CI) were calculated to assess the reproducibility of CGM results in 581 individuals. 62% were females 581 participants (62% women) mean age 48 years (range 18–87) were included, 12% had previously been diagnosed with diabetes. The inter-day reproducibility of the CGM results was greater for subjects with diabetes (ICC 0.46 [CI 0.39–0.55]) than for normoglycaemic subjects (ICC 0.30 [CI 0.27–0.33]); the value for prediabetic subjects was intermediate (ICC 0.37 [CI 0.31–0.42]). For normoglycaemic subjects, inter-day reproducibility was poorer among the younger (ICC 0.26 [CI 0.21–0.30]) than the older subjects (ICC 0.39 [CI 0.32–0.45]). Inter-day reproducibility was poorest among normoglycaemic subjects, especially younger normoglycaemic subjects, suggesting the need to monitor some patient groups more often than others.

## Introduction

In recent years, continuous glucose monitoring (CGM) systems have positioned themselves as a very useful tool to improve metabolic control in patients with diabetes. These devices allow obtaining more complete information on glycemic behavior than through traditional measurement methods (capillary blood glucose), allowing the patient and the health professional to make more complex therapeutic decisions that have an impact on improving metabolic control^1^. We currently have a growing number of product options for using CGM which we can classify into (1) real-time CGM systems (rtCGM) (2) intermittently scanned CGM systems (isCGM) and (3) professional CGM systems. All these devices measure interstitial glucose levels and provide numerical and graphical information about glucose profiles however there are differences with respect to handling and clinical use. rtCGM system send glucose information continuously from the sensor to the user’s receiver, smartphone, or insulin pump. isCGM systems provide and store the information when the user approaches the receiver or smartphone to the sensor. Professional CGM refers to use of devices that are owned by the clinic and used to retrospectively analyze glucose data. These devices can be used in the “blinded” mode to capture information about what patients are doing without influencing their behavior^2^.

Since their introduction, these devices have revolutionized^3^ our understanding of glycemic behaviour, enabling more precise monitoring than ever before. With their impressive technical capabilities, CGM systems hold tremendous potential for both clinical and research applications. One of the most significant advantages of CGM systems is their ability to enhance glycemic control. By providing real-time glucose data, these devices may help a larger number of patients achieve and maintain glycemic target glycated hemoglobin and time in range values^4^ while minimizing the risk of hypoglucemia^5^. Furthermore, the reliability and accuracy of CGM systems have paved the way for seamless integration with subcutaneous insulin infusion systems. This integration allows insulin delivery to be dynamically adjusted based on CGM information, offering an automated and efficient approach to manage blood glucose levels^6–8^.

CGM have shown promise in not only monitoring glucose levels in patients with diabetes but also in epidemiological studies evolving healthy volunteers and the general population^9–11^. These studies have provided valuable insights into glycemic behaviour of health individuals under real-life conditions. Understanding glucose profiles in non-diabetic populations has significant clinical implications, ranging from detecting early dysglycemia to preventing or delaying the onset of diabetes. Additionally, investigating post-prandial responses to nutrient combinations is of great interest at improving the overall health of the general population^12^.

To ensure proper utilizations of CGMs, it is crucial to assess the reliability and reproducibility of these devices over multiple days, considering the biological variations across individuals and time (see an example in another domain^13^). This evaluation is necessary to understand how perform in different populations and to stablich effective recommendations for clinical decisions derived from CGM procedures.

In a near future, with the development in the material, power supply, and data transmission area, the wearable point-of-care glucose sensors will be more miniaturized, accurate, and self-powered^14^. The success of CGM technology will depend on the quality of the monitoring information it provides. It is essential that the validity and reliability of the measurements taken be known. Traditionally, validity has been determined by comparing the values obtained by monitoring devices to standard reference values obtained by the self‐monitoring of capillary blood glucose^6^. Reliability or reproducibility refers to the consistency of measurements across repeated tests and/or varying conditions^15^; with respect to CGM systems, this has not been addressed.

Subcutaneous CGM sensors measure the glucose concentration of the interstitial space and use algorithms to extrapolate blood glucose levels from the values recorded^16^. In devices that display glucose values every 5 min, 288 values are reported each day. However, understanding glycaemic behaviour requires a knowledge of the magnitude of fluctuations in blood sugar concentration, and an overall comprehension of its variability. The latter can be appreciated in the shape of blood glucose curves^17^.

Recent advances in statistical methods, such as functional data analysis (FDA)^18, 19^, have opened up valuable opportunities to gain deeper insights into the complexities of glucose time series data obtained from CGM systems. FDA represents a powerful extension of traditional multivariate analysis which allows to treat mass data as dynamic curves that evolve over time. By treating the data as a collection of latent temporal processes. FDA facilitates the investigation of dynamic changes over time, providing a richer understanding of glucose dynamics. This novel approach holds significant potential for enhancing various statistical modeling techniques, including hypothesis testing and regression^19^. The utilization of FDA can introduce greater accuracy and reliability in predicting clinical outcomes or detecting relevant clinical and statistical differences related to glucose fluctuations.

To date, no study has used FDA to examine the reproducibility of results provided by CGM systems. The present work uses an FDA method to examine those thus obtained in a random sample of a free-living, adult population, with respect to its members' glycaemic status.

## Materials and methods

### Study design

The study subjects in this cross-sectional investigation were a subset of those enrolled in the A Estrada Glycation and Inflammation Study (AEGIS), trial NCT01796184 at www.clinicaltrials.gov. A detailed description of the latter study has been published elsewhere^10^. AEGIS was a cross-sectional study conducted in the municipality of A-Estrada, in the northwest of Spain. An age-stratified random sample of the population aged 18 years and older was drawn from Spain’s National Health System Registry.

### Patients

Of the total of 1516 participants recruited a subsample of 622 individuals participated in the Glycation project, which included continuous glucose monitoring procedures. From March 2013 until March 2015, subjects were successively convened at the A Estrada Primary Care Centre (A Estrada, Galicia, Spain) where they (i) completed an interviewer-administered structured questionnaire that collected demographic and anthropometric data, (ii) provided a description of their lifestyle, including information on diet, physical activity, alcohol consumption and smoking, (iii) provided a fasting venous blood sample, and (iv) were prepared for CGM. Of the 622 subjects who consented to undergo a 6-day period of CGM (361 women, 220 men), 581 completed at least 2 days of monitoring; the data of these latter subjects were used in analyses. The remaining 41 subjects were excluded due to non-compliance with the demands of the protocol (n = 4), or difficulties in handling the device (n = 37).

Calculation of body mass index. Body mass index (BMI) was calculated as body weight (kg)/height (m)^2^.

Assessment of physical activity. Subjects completed the International Physical Activity Questionnaire (short form), and the metabolic equivalents of the hours per week engaged in vigorous and moderate activities and walking were calculated as described by Craig^20^. Subjects were then classified as either (i) "inactive" (n = 209, 36%), (ii) "minimally active" (n = 223, 38%), or (iii) HEPA (health-enhancing physical activity)-active" (n = 149, 26%).

Classification of alcohol consumption and smoking. Alcohol consumption was recorded in terms of standard drinking units, summing the number of glasses of wine (~ 10 g alcohol per glass), bottles of beer (~ 10 g per 200 ml), and units of spirits (~ 20 g alcohol per measure) regularly consumed per week, as previously described^21^. Smoking habits were recorded as the number of cigarettes regularly consumed per day. Consumers of at least one cigarette per day were considered smokers. Individuals who had quit smoking during the preceding year were regarded as smokers.

Baseline and final laboratory determinations. Glucose was determined in plasma samples from fasting participants via the glucose oxidase peroxidase method. Glycated haemoglobin (A1C) was determined by high-performance liquid chromatography using a Menarini Diagnostics HA-8160 analyser; all A1C values were converted to DCCT-aligned values^22^. Serum insulin was determined using the ADVIA Centaur XP immunoassay system (Siemens, Erlangen, Germany). Insulin resistance was estimated using the homeostasis model assessment method (HOMA-IR) as the fasting concentration of plasma insulin (µ units/mL) × plasma glucose (mg/dL)/405^23^.

Baseline glycaemic status. Individuals were deemed to be diabetic if they had been previously diagnosed as such, or had an A1C level of > 6.4%, and/or a fasting plasma glucose (FPG) concentration of > 125 mg/dL (n = 70, 12%). Subjects with prediabetes were defined as those with an A1C range of 5.7–6.4% or a FPG range of 100–125 mg/dL (n = 121, 21%). Normoglycaemic subjects were defined as those with an A1C of < 5.7% and an FPG of < 100 mg/dL (n = 390, 67%). Baseline glycemic status (normoglycemia, prediabetes, and diabetes) were defined according to the American Diabetes Association criteria^24^.

### Dietary variables

During the CGM assessment period (see below), participants monitored their food and drink intakes. They were instructed to record the weight or portion size of all items consumed, and to provide a detailed description of each at the time of consumption. At the end of each subjects' assessment period, a research dietician reviewed the intake records and asked for additional data when records appeared incomplete or implausible. Total energy intake (kcal), and intakes (g and %) of fat, protein, carbohydrate, sugar and fibre were determined using Dietowin^®^ 8.0 software (BioLogica Tecnologica Medica SL, Barcelona, Spain, http://www.bl-biologica.es/dietowin.htm), and mean daily totals calculated for each complete 24 h (midnight to midnight) period.

### CGM data collection

At the start of the patient monitoring period, a research nurse inserted an Enlite™ sensor (Medtronic Inc., Northridge, CA, USA) subcutaneously in the subject's abdomen, and instructed the subject in the care of the connected iPro™ model CGM device (Medtronic Inc, Northridge, CA, USA). iPro™ is a professional blind CGM system which provides retrospective information on glucose profiles. As the system does not provide data on glucose values during the use of the device, the behavior of the patients is not affected by the information from the CGM. The sensor continuously measures the glucose concentration of the interstitial space in the subcutaneous tissue, recording values (range 40–400 mg/dL) every 5 min, and storing them in the CGM device. On the 7th day the sensor was removed and the data downloaded for analysis, disregarding the first day's results. In addition, if data-acquisition failure totalled more than 2 h in a day, the entire day's data were discarded.

Subjects were also provided with a conventional OneTouchR VerioR Pro glucometer (LifeScan, Milpitas, CA, USA) and compatible lancets and test strips. To guarantee the reliability and quality of the monitoring data, the participants were instructed to perform at least 3 capillary blood glucose measurements per day (usually before the main meals). The capillary blood glucose readings were used to calibrate the iPro™ CGM system. Data from monitoring that could not be calibrated with at least 3 capillary blood glucose controls per day were excluded from the analysis.

For all 583 subjects, there were a total of 9980 paired points (median 18 samples per patient) with both capillary BG measurements and CGM system interstitial fluid glucose measurements. Overall sensor accuracy expressed as Mean Absolute Relative Difference (MARD) was 7.9%. The sensor accuracy was highest during periods of euglycemia and hyperglycemia (> 180 mg/dL) and lowest during hypoglycemia. The mean ARD was 7.8% when blood glucose was between 70 and 180 mg/dL; 9.5% when blood glucose was greater than 180 mg/dl; and 29.2% when blood glucose was less than 70 mg/dL. Eighty-seven percent of the device results were within 15 mg/dL of the capillary BG results (for results of less than 100 mg/dL), and 87% were within 15% of the capillary BG results (for results higher than 100 mg/dL). The performance of the system on the first day was different to that on the following days. MARDs for all capillary-sensor glucose paired points stratified by day (1–6) were 12.1%, 7.6%, 7.0%, 7.1%, 7.3% and 6.6%, respectively.

Thus, data from day 1 were excluded.

### Ethical issues

Written informed consent was obtained from all participants. The study was approved by the Regional Ethics Committee (Comité Ético de Investigación Clínica de Galicia, registration code: 2012/025) and conformed to the current Helsinki Declaration.

### Statistical analyses

To assess the inter-day reproducibility of the CGM results according to subject glycaemic status (normoglycaemic, prediabetic and diabetic), functional intraclass correlation coefficients (ICC) and their 95% confidence intervals (95% CI), were calculated. ICC coefficients range from 0 to 1, and can be interpreted as follows: < 0.4 poor agreement; 0.4–0.59 fair agreement; 0.60–0.74 good agreement; > 0.74 excellent agreement^25^. The functional iCC was computed using the instrumental methodology of a two-way ANOVA multilevel functional model, as introduced in reference^26^. To achieve this, we use a novel bootstrap methodology, which is elaborated on in a subsequent paper^27^. For those interested in ICC calculation for other study designs from a functional perspective, additional information can be found in reference^19^.

To provide an idea of the variability in glucose measurements recorded by the CGM, two measures of glycaemic variability were analyzed: the coefficient of variation (CV), a measure of intra-day glycaemic variability^28^ and the mean of the daily differences (MODD), a measure of inter-day glycaemic variability, as previously described^27^. Specifically, CV was calculated as 100 × standard deviation/mean glucose. And MODD was calculated as follows:

$$MODD=\frac{{\sum }_{t1}^{tk}\left|BGt-BGt-1440\right|}{k}$$

where *BG* is blood glucose at time *t*, and *k* is the number of observations where there is an observation at the same time 24 h (1440 min) ago.

For the normoglycemic subjects, ICC, CV and MODD were calculated stratifying by demographics (gender, age groups) and life-styles (tobacco and alcohol consumption, physical activity and diet). All statistical functional data analyses were conducted using the statistical software R. For the functional exploratory analysis, we employed the refund package. Visualizations of functional data were created using standard R plotting functions. Lastly, the estimation of the functional ICC calculation was performed using the R package I2C2.

The I2C2 procedure allows the determination of ICCs when working with complex datasets. A non-parametric bootstrap procedure was performed to determine the I2C2 confidence level^27^. The code for calculating functional ICC is available at http://www.biostat.jhsph.edu/~ccrainic/software.html, or https://neuroconductor.org/package/I2C2.

## Results

The subjects had a mean age of 48 years (range 18–87); 62% were females. Seventy (12%) had previously been diagnosed with diabetes (100% type 2), and 121 (21%) fulfilled the criteria of prediabetes. Among those with diabetes, 69% took oral antidiabetic medication, 4% used insulin alone, 17% used insulin plus oral drugs, and 10% used neither insulin nor oral antidiabetic drugs. None of the participants was on treatment with GLP1 analogue.

The subjects with diabetes were older than those with prediabetes, and also older than the normoglycaemic subjects (61 ± 12, 57 ± 12, and 43 ± 13 years; respectively). The subjects with diabetes were also more commonly men (50%, 40% and 36%; respectively). Subjects with diabetes and prediabetes had higher BMIs than their normoglycaemic counterparts 31.2 ± 5.1 and 31.1 ± 4.8 vs. 26.7 ± 4.6 kg/m^2^). As expected, baseline PFG, A1C and the HOMA-IR value, as well as the glycaemic variability indices (CV and MODD), were highest among subjects with diabetes (Table 1).

**Table 1:** Clinical characteristics of the study participants according to glycaemic status.

	Normoglycaemia (n = 390)	Prediabetes (n = 121)	Diabetes (n = 70)
Males, n (%)	137 (35)	48 (40)	35 (50)
Age, years	43 (13)	57 (12)	61 (12)
BMI, kg/m^2^	26.7 (4.6)	31.1 (4.8)	31.2 (5.1)
FPG, mg/dL	84 (8)	100 (10)	134 (35)
A1C, %	5.2 (0.2)	5.7 (0.3)	7.1 (1.2)
HOMA-IR, mg/dL × µUI/mL	2.2 (1.1)	4.2 (2.7)	7.2 (9.7)
CV, %	14.3 (4.0)	15.6 (4.8)	23.6 (6.0)
MODD	0.68 (0.20)	0.77 (0.25)	1.47 (0.88)

Data are expressed as means (SD). *BMI* body mass index, *FPG* fasting plasma glucose, *A1C* glycated haemoglobin, *HOMA-IR* homeostasis model assessment-insulin resistance, *CV* coefficient of variation, *MODD* mean of daily differences.

The inter-day reproducibility of the GCM measurements was greater in subjects with diabetes (ICC 0.46 [95% CI 0.39–0.55), than in either the normoglycaemic subjects (ICC 0.30 [95% CI 0.27–0.33]) or those with prediabetes (ICC 0.37 [95% CI 0.31–0.42]).

Table 2 shows the reproducibility (ICC) and glycaemic variability (CV and MODD) coefficients (and their 95% CI) for the normoglycaemic subjects in relation to demographic data and lifestyle.

**Table 2:** Reproducibility of continuous glucose monitoring records and glycaemic variability indices in normoglycaemic subjects according to demographic data and lifestyle.

	n (%)	Reproducibility	Glycaemic variability	
		ICC (95% CI)	CV (95% CI)	MODD (95% CI)
Gender				
Females	137 (35)	0.31 (0.28, 0.34)	14.5 (14.0, 15.0)	0.68 (0.66, 0.71)
Males	253 (65)	0.28 (0.24, 0.32)	13.9 (13.3, 14.6)	0.68 (0.66, 0.71)
Age group (years)				
18–39	160 (41)	0.26 (0.21, 0.30)	14.2 (13.6, 14.8)	0.69 (0.66, 0.72)
40–59	180 (46)	0.29 (0.26, 0.36)	14.4 (13.8, 15.0)	0.67 (0.64, 0.70)
60–81	50 (13)	0.39 (0.32, 0.45)	14.1 (12.8, 15.3)	0.68 (0.62, 0.74)
Body mass index				
Normal weight	149 (38)	0.28 (0.23, 0.32)	14.6 (14.0, 15.3)	0.69 (0.65, 0.72)
Overweight	151 (39)	0.31 (0.27, 0.36)	14.2 (13.6, 14.9)	0.67 (0.64, 0.70)
Obese	90 (23)	0.28 (0.23, 0.33)	13.8 (13.0, 14.7)	0.69 (0.65, 0.74)
Alcohol consumption				
Abstainers	153 (39)	0.31 (0.27, 0.34)	14.3 (13.7, 14.9)	0.67 (0.64, 0.70)
Light drinkers	197 (51)	0.27 (0.23, 0.31)	14.6 (13.9, 15.1)	0.71 (0.68, 0.73)
Heavy drinkers	40 (10)	0.37 (0.29, 0.45)	12.9 (11.8, 14.1)	0.61 (0.55, 0.67)
Smoking				
Non-smokers	197 (50)	0.28 (0.24–0.31)	14.7 (14.1, 15.2)	0.70 (0.67, 0.73)
Ex-smokers	97 (25)	0.36 (0.31–0.40)	13.8 (12.9, 14.6)	0.64 (0.60, 0.67)
Smokers	96 (25)	0.29 (0.23–0.34)	14.0 (13.9, 14.7)	0.69 (0.65, 0.73)
Physical activity				
Inactive	138 (35)	0.29 (0.25–0.33)	14.6 (13.9, 15.3)	0.70 (0.66, 0.73)
Minimally active	145 (37)	0.32 (0.28–0.36)	14.1 (13.5, 14.8)	0.68 (0.64, 0.71)
HEPA	107 (28)	0.27 (0.22–0.31)	14.0 (13.3, 14.7)	0.67 (0.63, 0.71)
Daily number of meals				
2–3	245 (63)	0.31 (0.28–0.34)	14.5 (14.0, 15.0)	0.69 (0.66, 0.72)
4	101 (26)	0.27 (0.22–0.32)	14.1 (13.3, 14.9)	0.68 (0.64, 0.72)
5+	43 (11)	0.34 (0.28–0.39)	13.4 (12.5, 14.3)	0.64 (0.60, 0.68)
Calorie intake, kcal/day				
< 2000	174 (46)	0.31 (0.27–0.35)	15.0 (14.3, 15.6)	0.70 (0.68, 0.73)
2000–2500	118 (31)	0.29 (0.25–0.35)	13.8 (13.1, 14.5)	0.66 (0.62, 0.70)
2500+	87 (23)	0.28 (0.23–0.33)	13.8 (13.0, 14.6)	0.67 (0.62, 0.71)
Carbohydrate intake, %				
< 45	85 (22)	0.28 (0.23–0.33)	14.4 (13.5, 15.3)	0.68 (0.64, 0.73)
45–54	242 (62)	0.30 (0.27–0.34)	14.4 (13.9, 14.9)	0.68 (0.66, 0.71)
≥ 55	52 (13)	0.30 (0.24–0.35)	14.0 (13.0, 15.0)	0.67 (0.61, 0.72)

*ICC* functional intraclass coefficient correlation, *95% CI* 95% confidence interval, *CV* coefficient of variation, *MODD* mean of daily differences. Individuals with an alcohol consumption of 1–140 g/week were considered light drinkers, and those with > 140 g/week heavy drinkers. Alcohol abstainers and very occasional alcohol drinkers were pooled in the same category. Normal weight, body mass index (BMI) < 25 kg/m^2^; overweight, BMI 25–30 kg/m^2^; obese, BMI > 30 kg/m^2^. *HEPA-active* a high activity class.

The inter-day reproducibility of the CGM data was better in the elderly than in the younger people, although age influenced neither intra-day nor inter-day glycaemic variability.

As can be seen in Table 2 inter-day glycaemic variability (MODD) was lower in heavy drinkers than in light drinkers. CV was also lower in heavy drinkers than in light drinkers but without reaching statistical significance. Ex-smokers showed higher reproducibility and lower inter-day glycaemic variability than non-smokers. No significant differences were found in reproducibility nor glycaemic variability with respect to gender, BMI, physical activity, the number of meals consumed per day, daily calorie intake or daily carbohydrate intake.

## Discussion

The use of CGM systems in non-diabetic populations has witnessed a surge in recent years, offering valuable insights into glycemic behavior. These devices extend their clinical implications beyond the traditional monitoring and control of diabetes, encompassing applications in diabetes prevention and diagnosis, reinforcement of healthy habits, and evaluating treatments. Some of these applications are still in the developmental phase, showing promising potential, particularly in screening for diabetes-related diseases.

Given the significance of these diverse applications, it becomes imperative to comprehensively understand essential aspects of CGM system behavior across different populations and situations. Evidence-based recommendations for the optimal use of CGM devices rely heavily on grasping factors such as reproducibility. Ensuring the reliability and consistency of CGM data across days and various time scales is crucial in substantiating the validity of findings and recommendations.

This paper presents a pioneering perspective on exploring reproducibility in CGM data from a functional data analysis (FDA) standpoint. The methodology utilized in this study can prove instrumental in conducting similar essential analyses with other wearables and biosensors, paving the way for advancements in personalized healthcare and disease management^3, 19^.

The present results show the inter-day reproducibility of the GCM readings to be greater for the subjects with diabetes than for those with prediabetes or those who were normoglycaemic (ICCs = 0.46, 0.37 and 0.30, respectively). Overall, the inter-day reproducibility of the CGM readings was poor, although in patients with diabetes it might be considered fair. Among the normoglycaemic subjects, inter-day reproducibility became greater with age.

Recent studies have analyzed the influence of glycemic variability (GV) as an independent risk factor in the long-term development of diabetes-related complications^29^. In addition, the recommendations on clinical endpoints for the interpretation of continuous glucose monitoring data suggest that parameters that measure GV be included. Among the many metrics that exist to quantify GV, CV is the most appropriate measure to identify short-term glycemic variability and levels for patients with diabetes should be kept below 36%^4^, although some studies recommend lower targets (< 33%) for patients treated with insulin to reduce the risk of hypoglycemia^30^. In our study, patients with diabetes presented values of glycemic variability within the control objectives. Since our study, the evaluation of continuous glucose monitoring (CGM) metrics with healthy populations has seen significant progress. Recent studies in healthy populations in large-scale cohorts from Israel, such as the work by Keshet et al.^11^, have corroborated our glucose variability estimations. These findings have opened up new opportunities to assess the reliability of CGM metrics in conjunction with other biomarkers.

**Figure 1:**
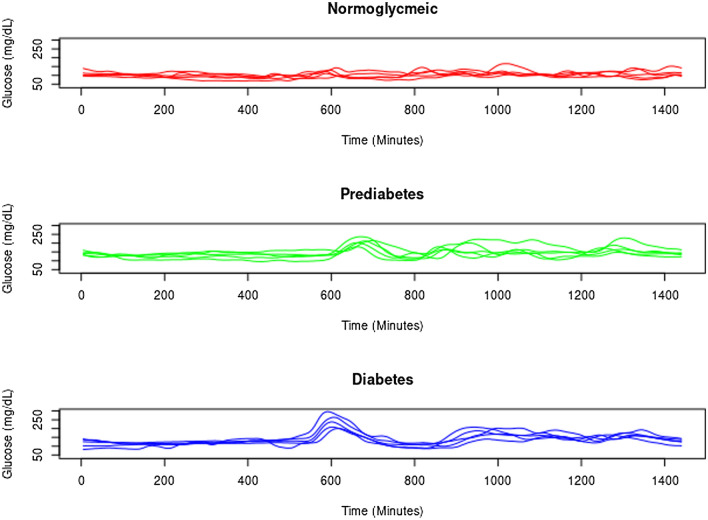
Continuous glucose monitoring profiles for 5 days in normoglycaemic subjects, and in those with prediabetes and diabetes.

Figure 1 shows different daily glucose profiles for the normoglycaemic, prediabetic and diabetic subjects. For the diabetic subjects, the curves for consecutive days appear to be more similar to one another, the possible result of these subjects showing less functional adaptation. This less complex dynamic behaviour means that future CGM readings are more predictable from preceding readings. Similarly, reproducibility increased with age in the normoglycaemic subjects, suggesting age to be associated with subjects showing less functional adaptation capacity and thus more stable CGM readings. In addition, the clinical recommendations in diabetes, regardless of the type of treatment and the degree of control, are based on maintaining a healthy eating plan adapted to pharmacological treatment, as well as carrying out a physical exercise plan. These recommendations can cause people to have a more organized way of life in daily life conditions regarding issues that affect the glycemic response (such as maintaining the proportion of carbohydrates and the timing of meals). However, more research is needed in different populations of patients with diabetes (for example, in individuals with high glycemic variability) and especially in people with prediabetes and normoglycemia.

The reproducibility of CGM readings was also greater among ex-smokers than non-smokers. This might be explained by the former being older. However, many smokers quitted because they had some smoking-related pathology which may have also reduced their functional adaptation capacity.

Different methods have been developed for analyzing the variability in physiological signals over time, and compared to non-diabetic subjects, reduced inter-day variability in blood glucose concentrations has been recorded in patients with diabetes^31, 32^, with a more pronounced loss of dynamic complexity in patients with type 1 diabetes^33^. Kohnert et al. also indicate beta-cell function to be an independent predictor of glucose time series dynamics as measured by detrended fluctuation analysis^34^, and report results compatible with increased glucose variability as determined from classical measures of standard deviation.

The AEGIS study database is one of the few population-based epidemiological studies using CGM technology in a random sample of a general population composed of normoglycemic, prediabetic, and diabetic patients. The use of continuous glucose monitoring in healthy populations^11, 35–39^ is topic of growing interest due to its multiple applications in epidemiological studies^17, 38^ or diet optimization^40, 41^. Therefore, our research is fundamental as it provides new information about the reproducibility of CGM with non-diabetic patients and uses the raw time series recorded by the CGM device as a richer piece of information.

The present results have important clinical implications. CGM monitoring over a single day may not be enough for solid conclusions on glucose homeostasis to be drawn; it may be necessary to monitor patients for longer. In February 2017, a panel of experts^42^ recommended a minimum of 14 consecutive days of CGM for optimal analysis and decision making a recommendation based on longitudinal CGM data from randomized trials undertaken by the The Juvenile Diabetes Research Foundation^43^. As expected, the fuller days of glucose data sampled, the stronger the correlation with 3 months' worth of data. The authors also suggested that a 12–15 day period of monitoring every 3 months may be needed to optimally assess overall glucose control. Unlike in the present study, the latter involved summary measures of glycaemic indices, and the correlation between different sample periods and the 3-month interval was determined via a coefficient based on ranks.

The amount and type of information provided by the CGM systems represent a challenge for its analysis, therefore it is necessary to develop new statistical methodologies^44^. Furthermore, we would like to emphasize that we are examining for the first time the reproducibility of CGM records with a raw analysis of temporal signals in the field of diabetes with techniques based on functional data analysis. This type of analysis provides a different and novel approach in the study of glycemic behavior through the information provided by glucose curves. The analysis of reliability with functional data analysis is a common technique with other devices such as Functional Magnetic Resonance Imaging (fMRI) or accelerometry devices^45, 46^. Traditional statistical methods may not adequately capture the dynamic nature of the CGM data and the underlying patterns. New statistical methods, such as functional data analysis (FDA), offer valuable insights into understanding the complexities of glucose time series data obtained from CGM systems. By analyzing the dynamic changes in glucose values over time, FDA provides a powerful extension of multivariate analysis. It allows us to explore the data as a set of latent temporal processes, represented, for example, by five glucose functions, from a functional perspective. This approach effectively exploits the underlying structure of the mathematical function in the data.

Functional data analysis may provide a potentially useful alternative approach^47, 48^. By considering the entire individual glucose trajectory as a functional unit and incorporating appropriate statistical models, functional data analysis may help researchers to characterize and compare glucose profiles, identify temporal patterns, and assess the impact of various factors on glucose dynamics^49, 50^. Incorporating FDA into the study of glucose time series data yields several modeling advantages, as highlighted in the biomechanics field^45^. For example, one hand, it enhances statistical power in hypothesis testing, enabling more robust and conclusive results. One another hand, FDA enables more accurate and precise regression modeling, providing a deeper understanding influence of the temporal dynamics in glucose levels.

This study suffers from the inherent limitations of its cross-sectional design, furthermore, the data were collected between March 2013 and March 2015 and CGM systems have advanced rapidly in the last 8 years. However, the sensor technology is the same (measurement of glucose oxidase in interstitial fluid) and the accuracy obtained (defined by its MARD) is similar to that presented in recent clinical studies^51^. The MARD is a parameter that expresses the average difference between the measurement of the system and the reference standard (in this case capillary blood glucose). Although the MARD is not as accurate when it is used to characterize the specific sensor alone, this parameter provides a number that reflects the total performance of a given CGM system in a clinical study^51^. Presentation of all MARD data available provide a better understanding of the CGM system performance and supports comparison of different CGM systems plus facilitates understanding the improvement seen with different generations of a given CGM system^51^.

In addition, no optimal number of monitoring days can be deduced. The strength of this work lies in its use of a new index to measure inter-day reproducibility that takes into account the dynamic nature of plasma glucose concentrations. The sample was also composed of subjects selected randomly from the general, free-living population, and included normoglycemic persons, those with prediabetes, and those with diabetes.

In conclusion, the present results show that CGM readings for diabetic subjects are more reproducible from one day to the next. This might indicate that these subjects have lost functional adaptational capacity compared to, for example, normoglycemic subjects. Given the promise of CGM systems as clinical tools, it would be of value to compile best practice guidelines aimed at increasing the reproducibility and validity of the results they provide.

## Data Availability

The datasets used and/or analysed during the current study available from the corresponding author on reasonable request.

## References

[1] Olczuk D, Priefer R (2018). A history of continuous glucose monitors (CGMs) in self-monitoring of diabetes mellitus. Diabetes Metab. Syndr..

[2] Kruger DF, Edelman SV, Hinnen DA, Parkin CG (2019). Reference guide for integrating continuous glucose monitoring into clinical practice. Diabetes Educ..

[3] Burge MR, Mitchell S, Sawyer A, Schade DS (2008). Continuous glucose monitoring: The future of diabetes management. Diabetes Spectr..

[4] Battelino T, Danne T, Bergenstal RM, Amiel SA, Beck R, Biester T, Bosi E, Buckingham BA, Cefalu WT, Close KL (2019). Clinical targets for continuous glucose monitoring data interpretation: Recommendations from the international consensus on time in range. Diabetes Care.

[5] Lin YK, Fisher SJ, Pop-Busui R (2020). Hypoglycemia unawareness and autonomic dysfunction in diabetes: Lessons learned and roles of diabetes technologies. J. Diabetes Investig..

[6] Choudhary, P., Kolassa, R., Keuthage, W., Kroeger, J., Thivolet, C., Evans, M., ADAPT study Group. Advanced hybrid closed loop therapy versus conventional treatment in adults with type 1 diabetes (ADAPT): A randomised controlled study. *Lancet Diabetes Endocrinol.* (2022). 10.1016/S2213-8587(22)00212-1 36058207

[7] Breton MD, Kanapka LG, Beck RW, Ekhlaspour L, Forlenza GP, Cengiz E (2020). A randomized trial of closed-loop control in children with type 1 diabetes. N. Engl. J. Med..

[8] Mariani HS, Layden BT, Aleppo G (2017). Continuous glucose monitoring: A perspective on its past, present, and future applications for diabetes management. Clin. Diabetes.

[9] Zhou J, Li H, Ran X, Yang W, Li Q, Peng Y (2011). Establishment of normal reference ranges for glycemic variability in Chinese subjects using continuous glucose monitoring. Med. Sci. Monit..

[10] Gude F, Díaz-Vidal P, Rúa-Pérez C, Alonso-Sampedro M, Fernandez-Merino C, Rey-Garcia J (2017). Glycemic variability and its association with demographics and lifestyles in a general adult population. J. Diabetes Sci. Technol..

[11] Keshet A, Shilo S, Godneva A (2023). CGMap: Characterizing continuous glucose monitor data in thousands of non-diabetic individuals. Cell Metab..

[12] Zeevi D, Korem T, Zmora N, Israeli D, Rothschild D, Weinberger A, Segal E (2015). Personalized nutrition by prediction of glycemic responses. Cell.

[13] Straczkiewicz, M., Keating, N. L., Thompson, E., Matulonis, U. A., Campos, S. M., Wright, A. A., & Onnela, J. P. Validation of an open-source smartphone step counting algorithm in clinical and non-clinical settings. *medRxiv*. 2023-03 (2023). 10.2196/47646 PMC10687676 37966891

[14] Zhang S, Zeng J, Wang C, Feng L, Song Z, Zhao W (2021). The application of wearable glucose sensors in point-of-care testing. Front. Bioeng. Biotechnol..

[15] Cronbach LJ, Wainer H, Braun H (1988). Five perspectives on validity argument. Test Validity.

[16] Kovatchev BP, Gonder-Frederick LA, Cox DJ, Clarke WL (2004). Evaluating the accuracy of continuous glucose-monitoring sensors: Continuous glucose-error grid analysis illustrated by TheraSense Freestyle Navigator data. Diabetes Care.

[17] Hall H, Perelman D, Breschi A, Limcaoco P, Kellogg R, McLaughlin T, Snyder M (2018). Glucotypes reveal new patterns of glucose dysregulation. PLoS Biol..

[18] Matabuena M, Vidal JC, Hayes PR, Saavedra-García M, Trillo FH (2019). Application of functional data analysis for the prediction of maximum heart rate. IEEE Access.

[19] Matabuena M, Karas M, Riazati S, Caplan N, Hayes PR (2023). Estimating knee movement patterns of recreational runners across training sessions using multilevel functional regression models. Am. Stat..

[20] Craig CL, Marshall AL, Sjostrom M, Bauman AE, Booth ML, Ainsworth BE (2003). International physical activity questionnaire: 12-country reliability and validity. Med. Sci. Sports Exerc..

[21] Gual A, Martos AR, Lligona A, Llopis JJ (1999). Does the concept of a standard drink apply to viticultural societies?. Alcohol Alcohol..

[22] Hoelzel W, Weykamp C, Jeppsson JO, Miedema K, Barr JR, Goodall Y (2004). IFCC Working Group on HbA1c Standardization: IFCC reference system for measurement of hemoglobin A1c in human blood and the national standardization schemes in the United States, Japan, and Sweden: a method-comparison study. Clin. Chem..

[23] Matthews DR, Hosker JP, Rudenski AS, Naylor BA, Treacher DF, Turner RC (1985). Homeostasis model assessment: Insulin resistance and beta-cell function from fasting plasma glucose and insulin concentrations in man. Diabetologia.

[24] American Diabetes Association (2020). Classification and diagnosis of diabetes: Standards of medical care in diabetes-2020. Diabetes Care.

[25] Cicchetti DV, Sparrow SA (1981). Developing criteria for establishing interrater reliability of specific items: Applications to assessment of adaptive behavior. Am. J. Ment. Def..

[26] Di CZ, Crainiceanu CM, Caffo BS, Punjabi NM (2009). Multilevel functional principal component analysis. Ann. Appl. Stat..

[27] Molnar GD, Taylor WF, Ho MM (1972). Day-to-day variation of continuously monitored glycaemia: A further measure of diabetic instability. Diabetologia.

[28] Service FJ (2013). Glucose variability. Diabetes.

[29] Piona C, Ventrici C, Marcovecchio L, Chiarelli F, Maffeis C, Bonfanti R (2021). Long-term complications of type 1 diabetes: What do we know and what do we need to understand?. Minerva Pediatr..

[30] Rama Chandran S, Tay WL, Lye WK, Lim LL, Ratnasingam J, Tan ATB, Gardner SLD (2018). Beyond HbA1c: Comparing glycemic variability and glycemic indices in predicting hypoglycemia in type 1 and type 2 diabetes. Diabetes Technol. Ther..

[31] Yamamoto N, Kubo Y, Ishizawa K, Kim G, Moriya T, Yamanouchi T (2010). Detrended fluctuation analysis is considered to be useful as a new indicator for short-term glucose complexity. Diabetes Technol. Ther..

[32] Khovanova NA, Khovanov IA, Shabno L, Griffiths F, Holt TA (2013). Characterisation of linear predictability and non-stationarity of subcutaneous glucose profiles. Comput. Methods Progr. Biomed..

[33] Kohnert KD, Heinke P, Vogt L, Augstein P, Salzsieder E (2018). Applications of variability analysis techniques for continuous glucose monitoring derived time series in diabetic patients. Front. Physiol..

[34] Kohnert KD, Heinke P, Vogt L, Augstein P, Salzsieder E (2014). Declining ß-cell function is associated with the lack of long-range negative correlations in glucose dynamics and increased glycemic variability: A retrospective analysis in patients with type 2 diabetes. J. Clin. Transl. Endocrinol..

[35] Hirsch IB, Armstrong D, Bergenstal RM, Buckingham B, Childs BP, Clarke WL (2008). Clinical application of emerging sensor technologies in diabetes management: Consensus guidelines for continuous glucose monitoring (CGM). Diabetes Technol. Ther..

[36] Holzer R, Bloch W, Brinkmann C (2022). Continuous glucose monitoring in healthy adults-possible applications in health care, wellness, and sports. Sensors.

[37] Klonoff DC, Nguyen KT, Xu NY, Gutierrez A, Espinoza JC, Alaina PV (2022). Use of continuous glucose monitors by people without diabetes: An idea whose time has come?. J. Diabetes Sci. Technol..

[38] Shah VN, DuBose SN, Li Z, Beck RW, Peters AL, Weinstock RS (2019). Continuous glucose monitoring profiles in healthy nondiabetic participants: A multicenter prospective study. J. Clin. Endocrinol. Metab..

[39] Pazos-Couselo M, Portos-Regueiro C, González-Rodríguez M (2022). Aging of glucose profiles in an adult population without diabetes. Diabetes Res. Clin. Pract..

[40] Ben-Yacov O, Godneva A, Rein M, Shilo S, Kolobkov D, Koren N (2021). Personalized postprandial glucose response-targeting diet versus mediterranean diet for glycemic control in prediabetes. Diabetes Care.

[41] Leshem A, Segal E, Elinav E (2020). The gut microbiome and individual-specific responses to diet. Msystems.

[42] Danne T, Nimri R, Battelino T, Bergenstal RM, Close KL, DeVries JH (2017). International consensus on use of continuous glucose monitoring. Diabetes Care.

[43] Xing D, Kollman C, Beck RW, Tamborlane WV, Laffel L, Buckingham BA (2011). Juvenile Diabetes Research Foundation Continuous Glucose Monitoring Study Group. Optimal sampling intervals to assess long-term glycemic control using continuous glucose monitoring. Diabetes Technol. Ther..

[44] Matabuena M, Petersen A, Vidal JC, Gude F (2021). Glucodensities: A new representation of glucose profiles using distributional data analysis. Stat. Methods Med. Res..

[45] Shou H, Eloyan A, Lee S, Zipunnikov V, Crainiceanu AN, Nebel NB (2013). Quantifying the reliability of image replication studies: The image intraclass correlation coefficient (I2C2). Cogn. Affect. Behav. Neurosci..

[46] Caceres A, Hall DL, Zelaya FO, Williams S, Mehta M (2009). Measuring FMRI reliability with the intra-class correlation coefficient. Neuroimage.

[47] Guo W (2002). Functional mixed effects models. Biometrics.

[48] Ferraty, F., Vieu, P. *Nonparametric Functional Data Analysis.* (Springer Series in Statistics, 2006).

[49] Gecili E, Huang R, Khoury JC, King E, Altaye M, Bowers K (2020). Functional data analysis and prediction tools for continuous glucose-monitoring studies. J. Clin. Transl. Sci..

[50] Mahmoudi Z, Del Favero S, Jacob P, Choudhary P, Consortium H-R (2021). Toward an optimal definition of hypoglycemia with continuous glucose monitoring. Comput. Methods Progr. Biomed..

[51] Heinemann L, Schoemaker M, Schmelzeisen-Redecker G, Hinzmann R, Kassab A, Freckmann G, Reiterer F, Del Re L (2020). Benefits and limitations of MARD as a performance parameter for continuous glucose monitoring in the interstitial space. J. Diabetes Sci. Technol..

